# Supplementary material for: Pipeline Olympics: continuable benchmarking of computational workflows for DNA methylation sequencing data against an experimental gold standard
Source: Nucleic Acids Res. 2025 Oct 21;53(19):gkaf970. doi: 10.1093/nar/gkaf970 (PMC12539629; doi:10.1093/nar/gkaf970)
Supplement: gkaf970_Supplemental_Files [file gkaf970_supplemental_files.zip › PipelineOlympics_Jul2025_Supplementary_Material.pdf]

Supplementary Material to

Pipeline Olympics: continuable benchmarking of computational workflows for DNA methylation sequencing data against an experimental gold standard

Yu-Yu Lin, Kersten Breuer, Dieter Weichenhan, Pascal Lafrenz, Antonella Sarnataro, Agata Wilk, Maryna Chepeleva, Oliver Mücke, Maximilian Schönung, Franziska Petermann, Philip Reiner Kensche, Lena Weiser, Frank Thommen, Gideon Giacomelli, Karl Nordstroem, Edahi Gonzalez-Avalos, Angelika Merkel, Helene Kretzmer, Jonas Fischer, Stephen Krämer, Murat Iskar, Stephan Wolf, Ivo Buchhalter, Manel Esteller, Chris Lawerenz, Sven Twardziok, Marc Zapatka, Volker Hovestadt, Matthias Schlesner, Marcel H. Schulz, Steve Hoffmann, Clarissa Gerhauser, Jörn Walter, Mark Hartmann, Daniel B. Lipka, Yassen Assenov, Christoph Bock, Christoph Plass, Reka Toth*, Pavlo Lutsik*

Supplementary Table Legends

Supplementary Table 1: Workflow information.

Sheet “Published workflows”: Overview of published bisulfite sequencing processing workflows including code URLs and publication DOIs, publication years, citation counts and other impact metrics, maintenance status etc. Sheet “Evaluated workflows”: Detailed information of 10 evaluated workflows. Sheet “Parameters”: Workflow execution parameters for each protocol. Sheet “Installation and Documentation”: Evaluation of deployment options, installation simplicity and the quality of documentation. Sheet “I&D Scoring Scheme”: installation and documentation features conversion to numeric scores.

Supplementary Table 2: Sequencing protocols.

Sheet “Sequencing protocols overview”: Overview of five sequencing protocols used in the study. Sheet “Basic statistics”: quantitative features of generated methylation sequencing data for each sample, including mean PHRED scores, percentage of methylated non-CpG cytosines, conversion rate and read length. Sequences of artificial adapter sequences, based on the characteristics of the protocols which were removed before the processing are given for each protocol. Sheet “Raw data overview”: read and base counts for each generated FASTQ file.

Supplementary Table 3: Preprocessing details.

Sheet “Read retention (counts)”: Numbers of reads retained after each workflow step. Sheet “Read retention (%”): same in percentages. Sheet “Global metrics”: basic processing statistics of each workflow execution, including the fraction of CpGs covered, mean and median coverage depth as well as mean and median CpG methylation.

Supplementary Table 4: Gold standard evaluation.

Overview of reference loci used for the experimental gold standard evaluation including locus identifier, genomic coordinates (hg19) and size, brief description, identifier, position and strand of the HumanMethylation450 probe used for the selection, locus sequence, relation to and identifier of the closest gene, relation to a CpG island and CpG content.

Supplementary Table 5: Final scoring.

Complete workflow scoring table across all sequencing protocols including genome-wide fraction of covered CpGs, depth of coverage, genome-wide similarity, consensus corridor deviation, runtime and maximal memory usage.

Supplementary Figures

Supplementary Figure 1

Schematic overview of protocol chemistries for whole-genome bisulfite sequencing (WGBS), Swift Bio's Accel-NGS (Swift), Tagmentation-based whole-genome bisulfite sequencing (T-WGBS), PBAT with random priming (PBAT), and NEB's NEBNext (EM-seq). The amount of DNA used in this study is specified next to each protocol name. These protocols can be categorized into three groups based on the input DNA amount, which is indicated beneath the protocol name. The color coding refers to native DNA (dark blue), cytosine-converted DNA (light blue), adaptors (orange and green) and unmethylated artificial ends (purple).

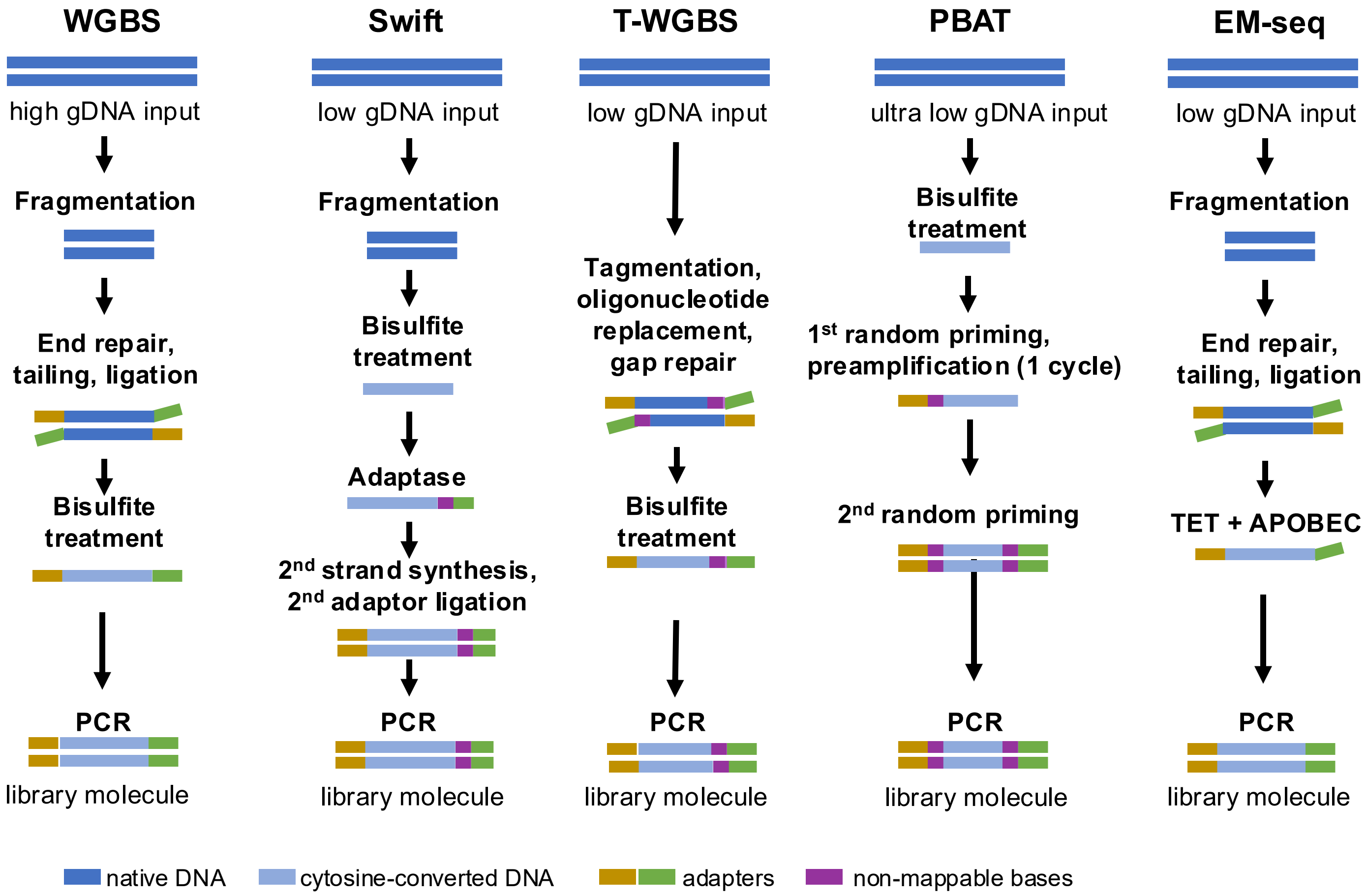

Supplementary Figure 1:

Supplementary Figure 2

Characteristics of methylation data obtained with different protocols. a. An extension of Figure 2a. The histogram displays the distribution of depth for the different protocols by samples. The boxplot at the bottom of the histogram indicates the median and interquartile range (IQR) values, offering insight into the central tendency and spread of the distribution. b. An extension of Figure 2b. The density distribution of the GC content of the reads by samples. Note that the read sequence itself was not used, but rather the reference genome sequence at the corresponding alignment position. c. M-bias plots show average methylation levels by read position for the samples named 5N, 6N (normal), and 5T, 6T (tumor), separately for read 1 and read 2. In the ideal case, the level of methylation is independent of the position. The read lengths vary between protocols due to differences in both trimming and the original read lengths.

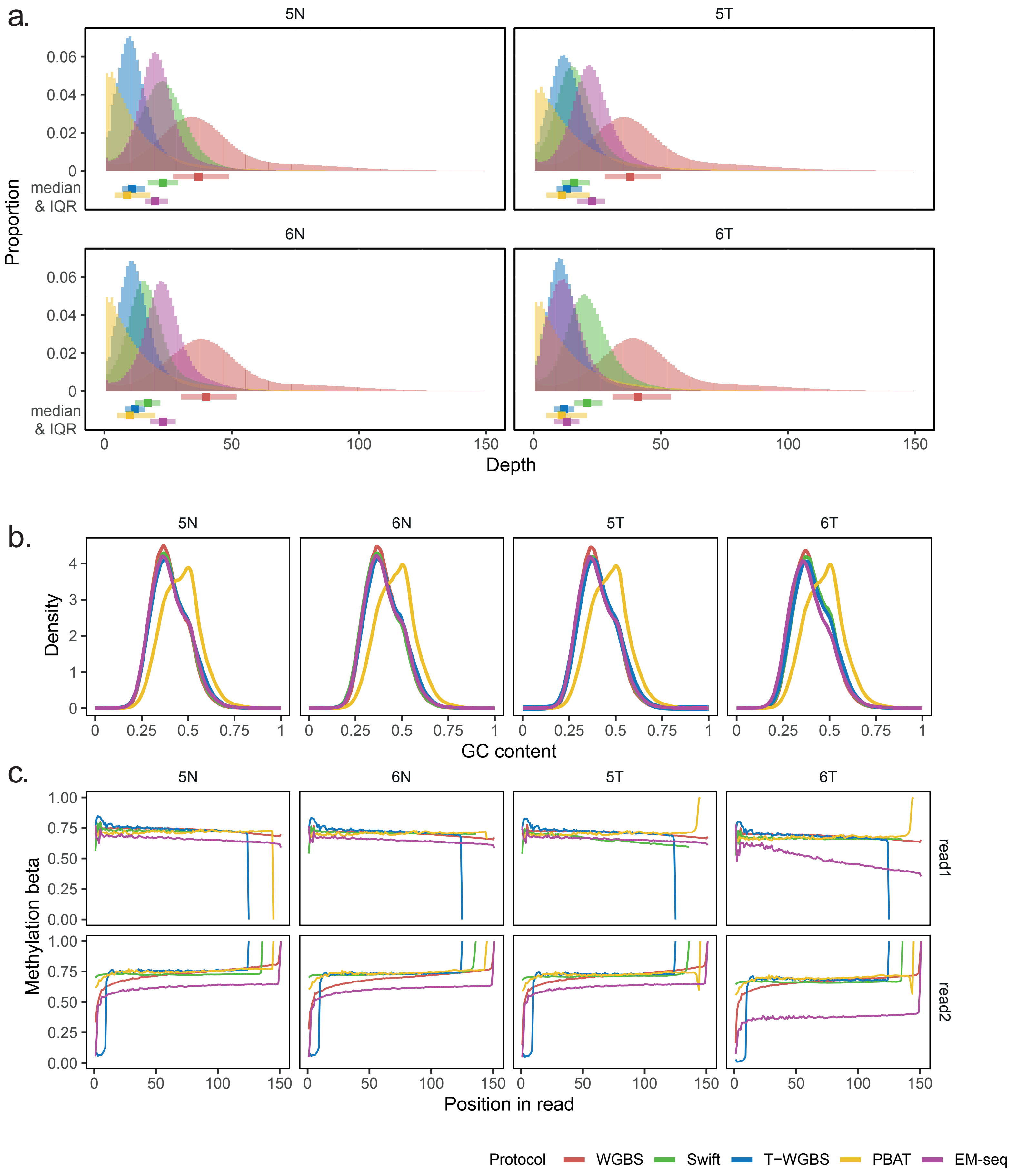

Supplementary Figure 2:

Supplementary Figure 3

Fractions of retained reads after the trimming, alignment, and duplicate removal steps. Workflow steps are given on the horizontal dimension. All fractions are calculated based on the read number of raw data. UNK indicates the workflow does not provide the corresponding BAM files. NA indicates the workflow failed on the dataset (BAT does not support PBAT and *gemBS* failed on PBAT) or does not include this step (BAT does not contain a deduplication step).

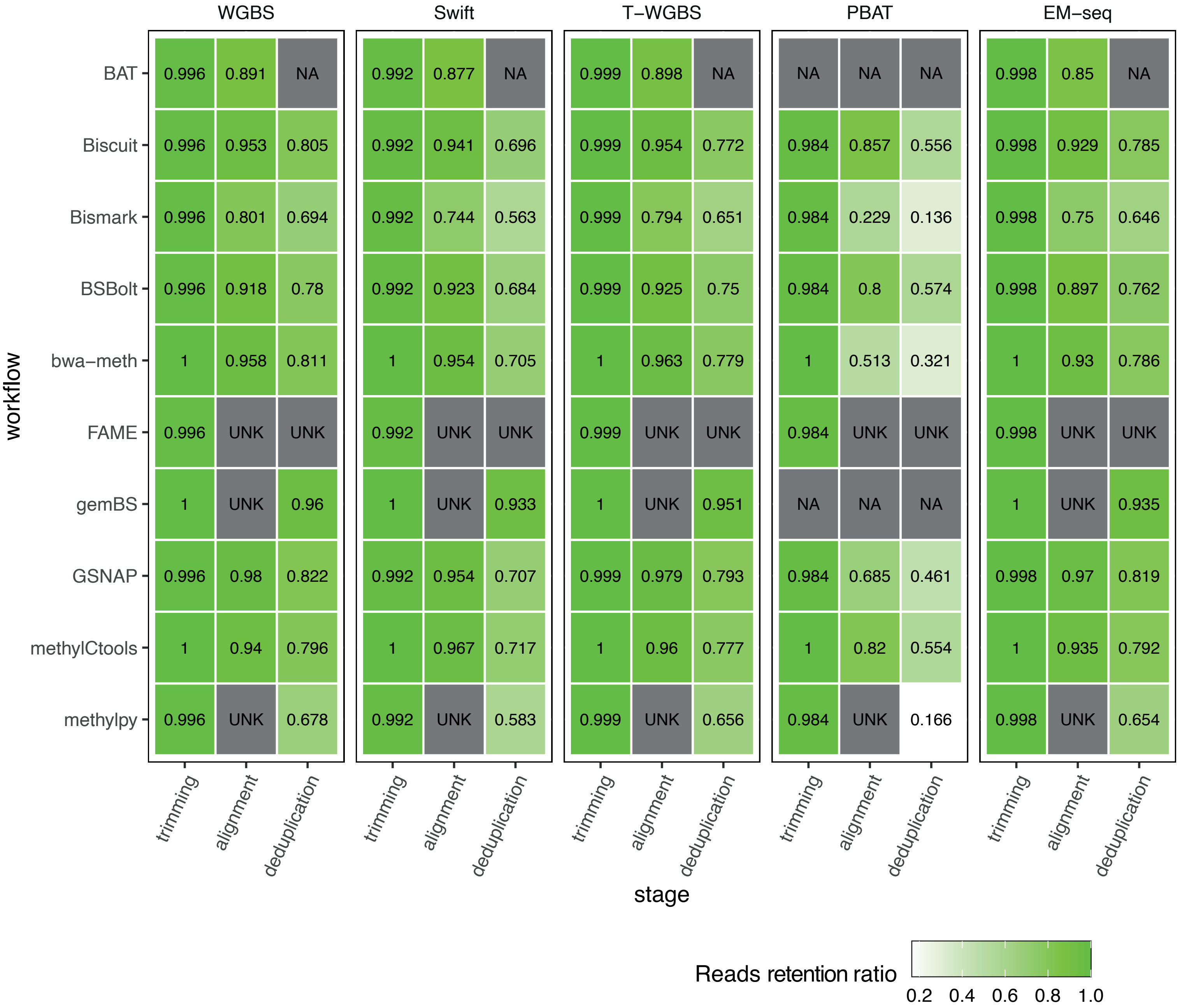

Supplementary Figure 3:

Supplementary Figure 4

Distribution of methylation values in WGBS data after downsampling to PBAT coverage (extension of Figure 3c). An accentuated mode at approximately 0.8 was observed in the beta distribution of WGBS. Unlike the other four protocols, which all display two peaks at 0 and 1, WGBS does not exhibit a distinct peak at 1. This phenomenon is suspected to be a result of the higher read coverage associated with WGBS, causing the peaks to shift from 1 towards 0.8. To verify our hypothesis, we downsized the WGBS sample to 8x read coverage (matching the depth of PBAT in the study), and the down-sampled reveals a peak at position 1, with a distribution closely resembling that of the other four protocols.

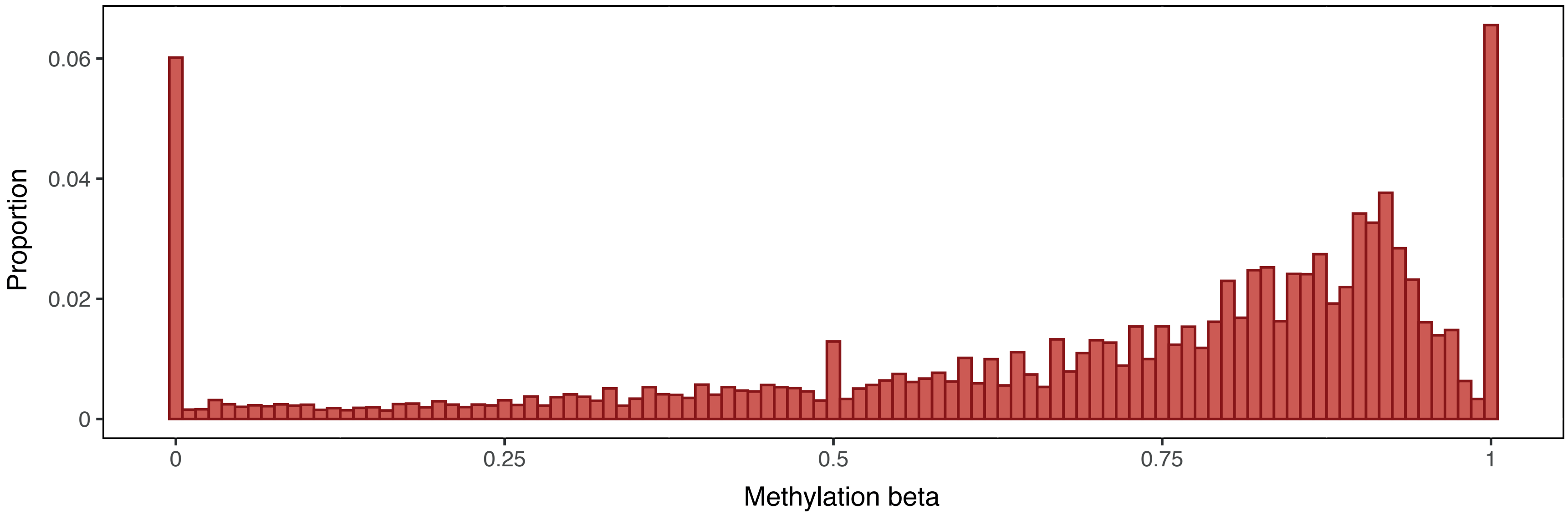

Supplementary Figure 4:

Supplementary Figure 5

Abundance of chimeric reads in PBAT libraries. We confirmed the presence of chimeric reads, as shown in previous studies, which contain sequences from two or more distinct genomic loci. To assess the occurrence of chimeric reads in different protocols, we define reads that map to different chromosomes as chimeric reads and calculate their proportion relative to the total mapped reads. This analysis reveals that the proportion of chimeric reads generated in PBAT is approximately 6.64 times that of Swift and 10.33 times that of WGBS. The Y-axis represents the fraction of chimeric reads among mapped reads. The counts for mapped reads and chimeric reads are extracted from the *samtools flagstat* report. In Line 5, 'mapped' is used to denote the number of mapped reads, and in Line 12, 'with mate mapped to a different chr' is used to represent the number of chimeric reads.

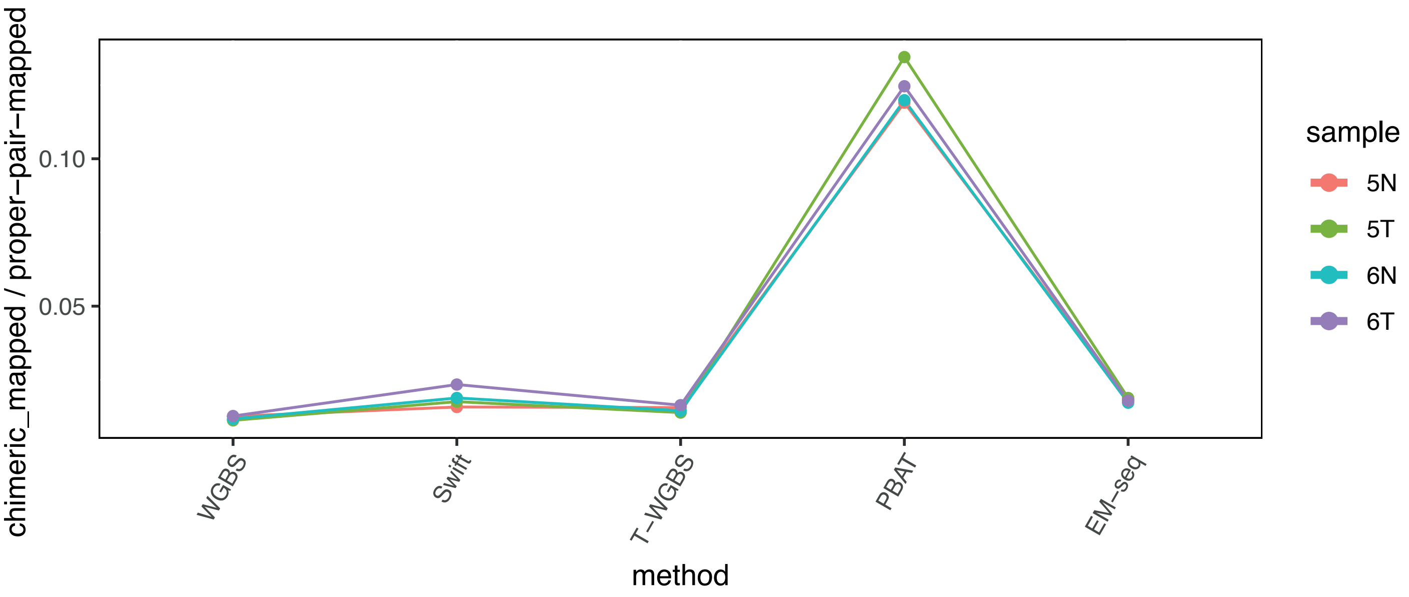

Supplementary Figure 5:

Supplementary Figure 6

Breadth vs depth of coverage diagrams (an extension of Figure 3a). These plots display the percentage of covered CpGs (y-axis) below varying read coverage thresholds in log10 scale (x-axis). It provides a practical method for determining the proper coverage cut-off threshold.

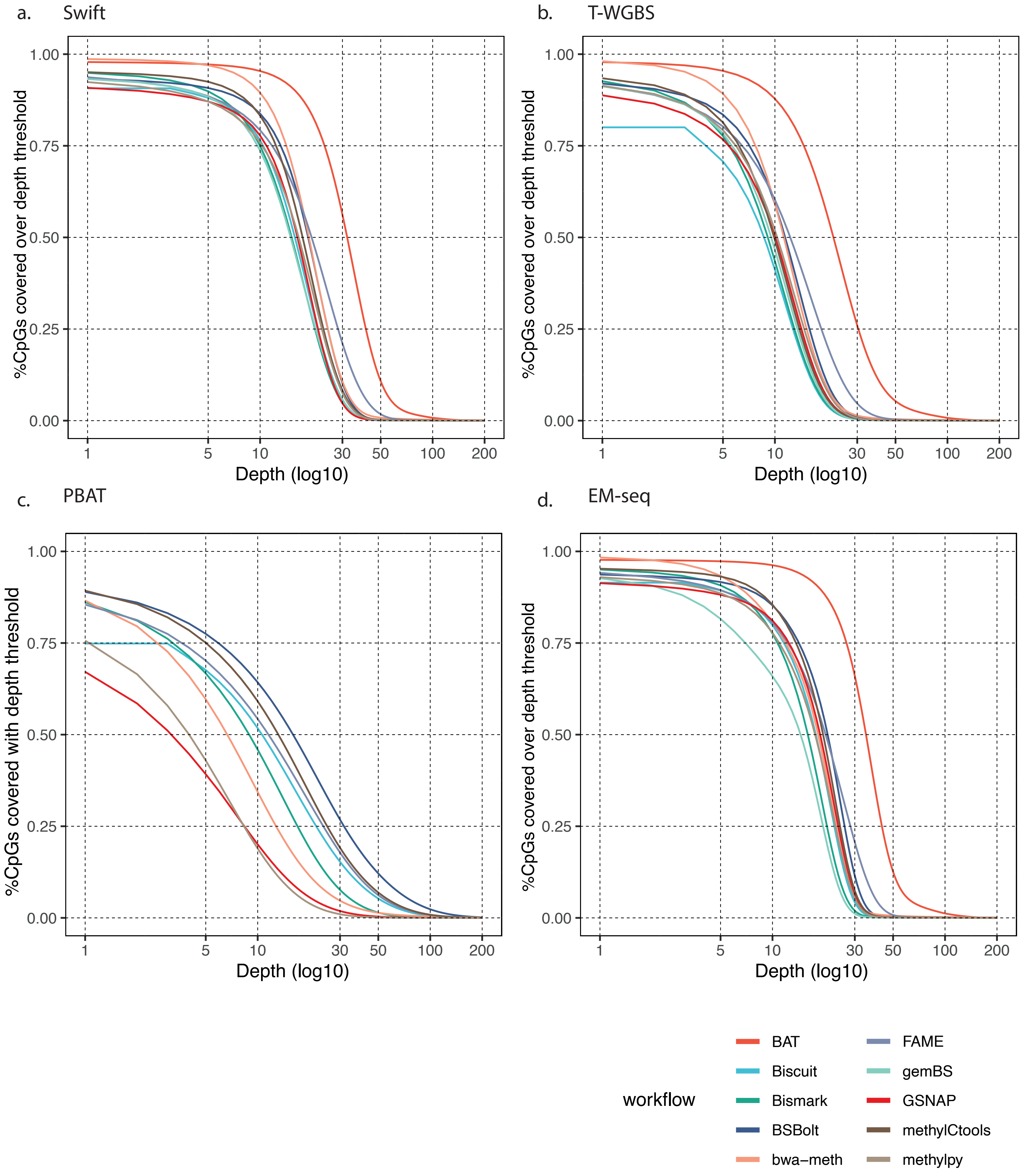

Supplementary Figure 6:

Supplementary Figure 7

The distribution of beta values for sample-protocol pairs. The beta value of each workflow is represented in a separate line on each plot.

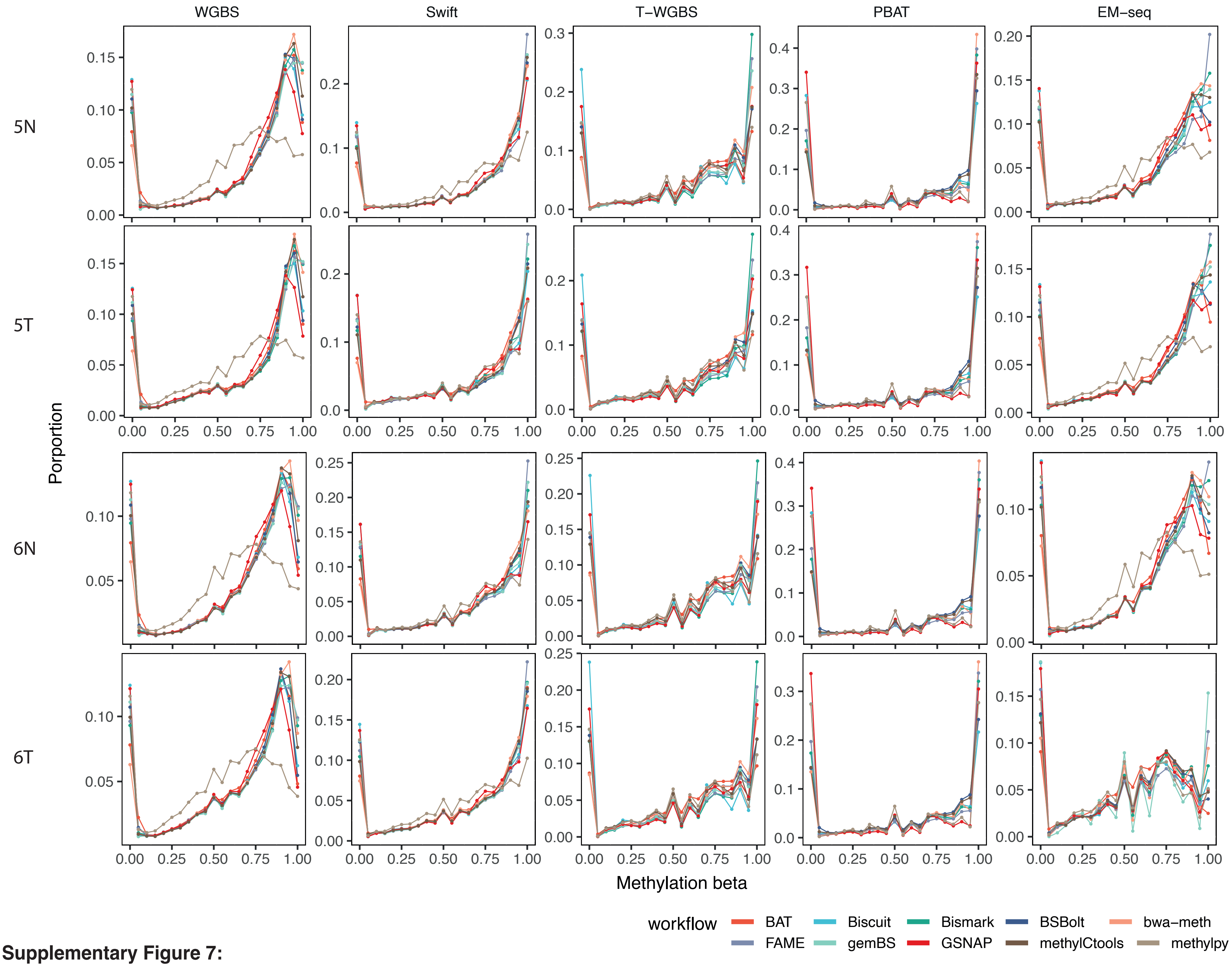

Supplementary Figure 7:

Supplementary Figure 8

Deviation stratified by classes of genomic regions. Hg38 annotation of R package *annotatr* (v1.14.0) was used. Mean deviation over all four samples is shown. The mean of the deviation among all workflows sorts the annotations (x-axis). The order of y-axis is the final ranking.

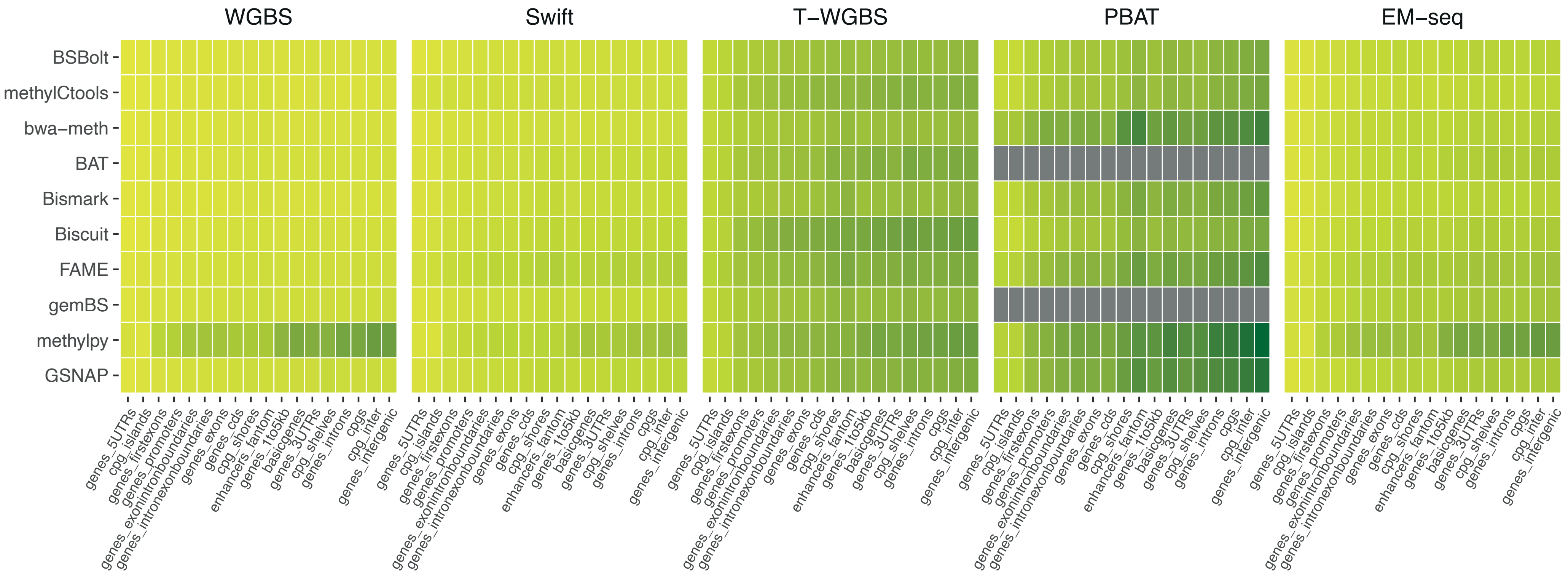

WGBS
Swift
T−WGBS
PBAT
EM−seq
BSBolt
methylCtools
bwa−meth
BAT
Bismark
Biscuit
FAME
gemBS
methylpy
GSNAP
genes_5UTRs
cpg_islands
genes_firstexons
genes_promoters
genes_exonintronboundaries
genes_intronexonboundaries
genes_exons
genes_cds
cpg_shores
enhancers_fantom
genes_1to5kb
basicgenes
genes_3UTRs
cpg_shelves
genes_introns
cpgs
cpg_inter
genes_intergenic
mean deviation
0.05
0.10
0.15

Supplementary Figure 9

Relationship between alignment rate and absolute means deviation on whole-genome scale in the PBAT dataset. Correlation suggests that a higher alignment rate is associated with greater accuracy, as indicated by a lower absolute mean deviation. The Spearman correlation and p-value on the plot represent the average of sample-wise calculations.

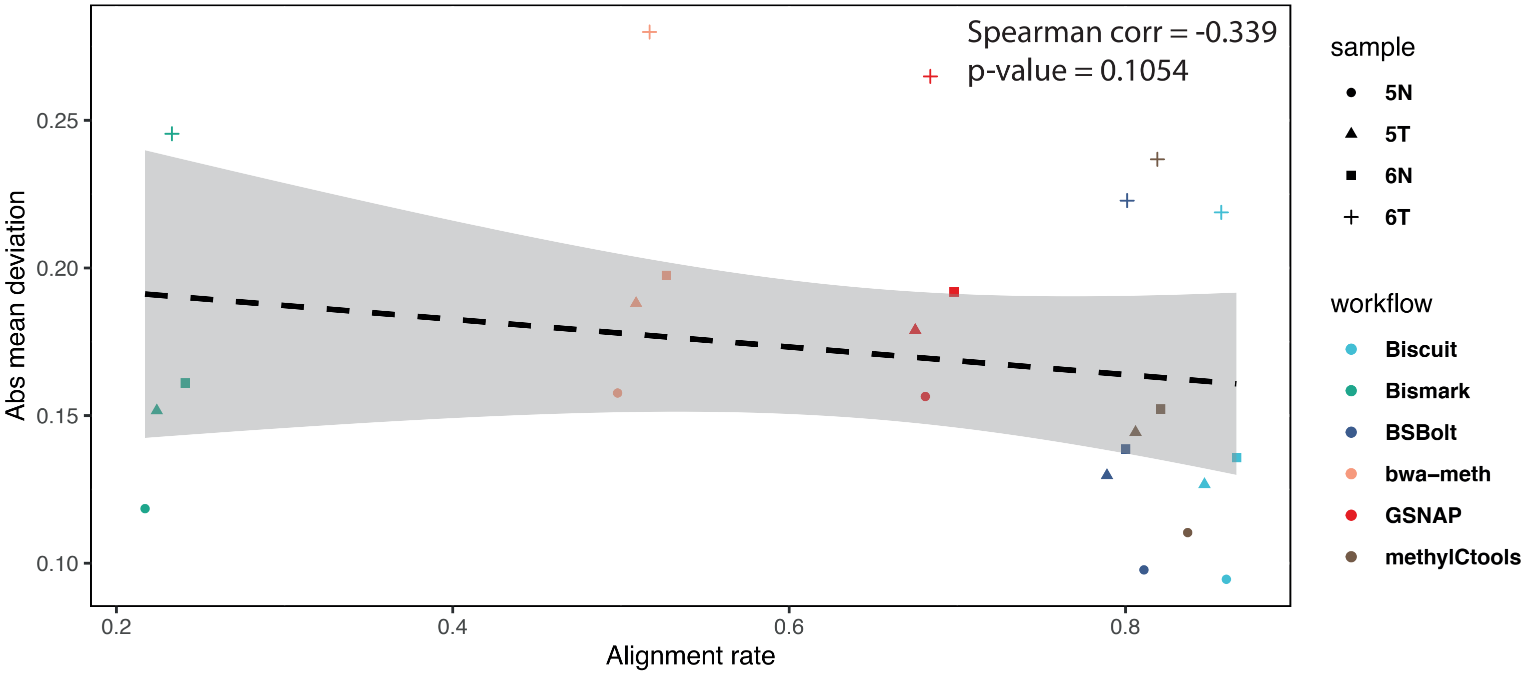

Supplementary Figure 9:

Supplementary Figure 10

Deviation plot of 46 loci (WGBS) for assessing gold standard-based methylation call accuracy. The plot displays the deviation from the gold-standard consensus corridors for these 46 selected loci. The grey boxes represent the consensus corridors, as reported by the BLUEPRINT Consortium. Each dot on the plot represents a measured beta value for a specific workflow, and the lines connecting the dots depict their respective deviations from the consensus corridors. The loci are sorted by the mean of the consensus corridor. This plot serves as an extension of Figure 5a, which only indicates 6 loci.

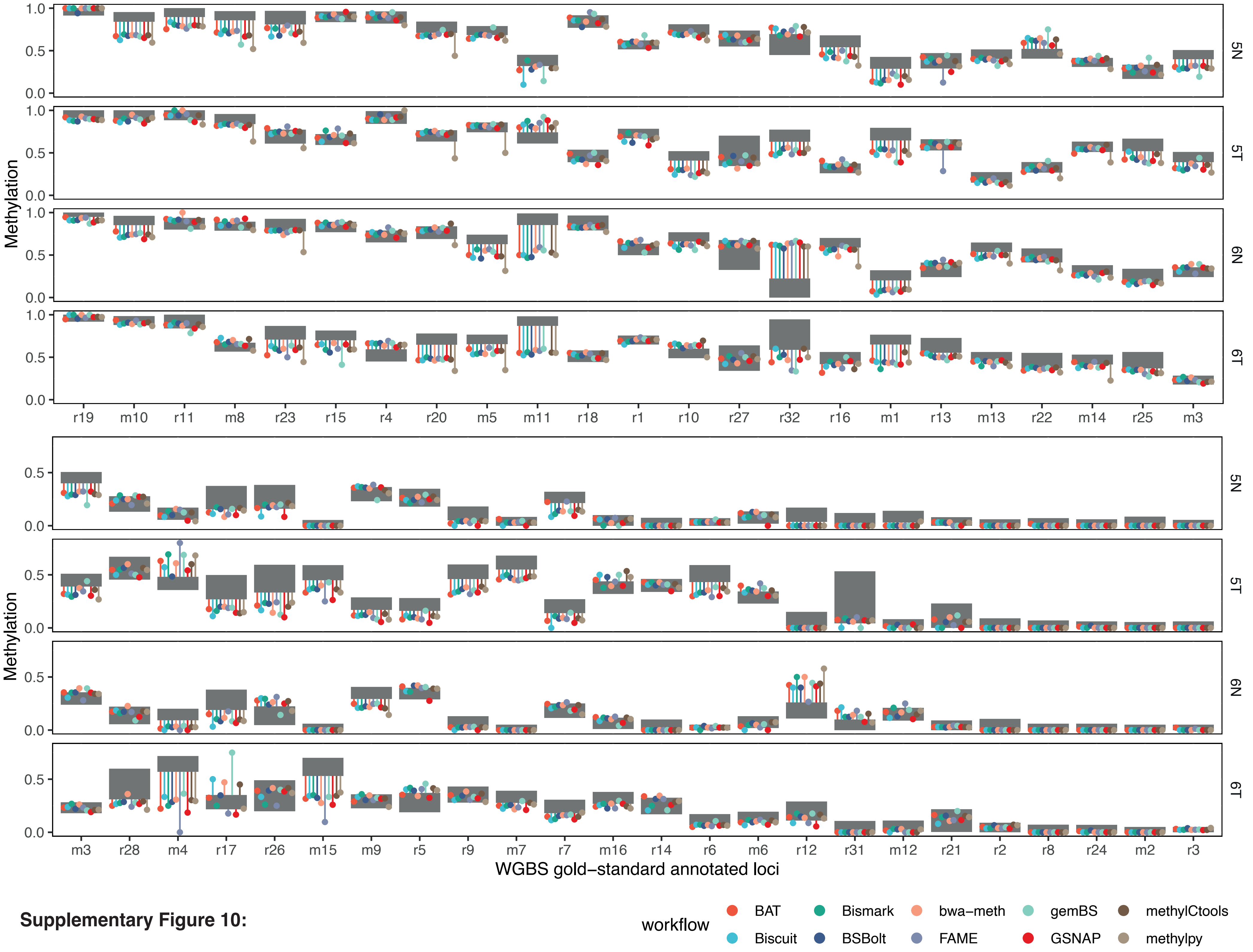

Supplementary Figure 10:

Supplementary Figure 11

Same as Supplementary Figure 10 but using down-sampled WGBS alignments to a coverage level equivalent to PBAT (8x coverage) to assess the effect of read coverage. We excluded workflows that were not run on the PBAT dataset and those that did not provide BAM files. As a result, this plot involves only 6 workflows. It represents the deviation of 46 loci in the down-sampled WGBS dataset, which now matches the coverage of PBAT.

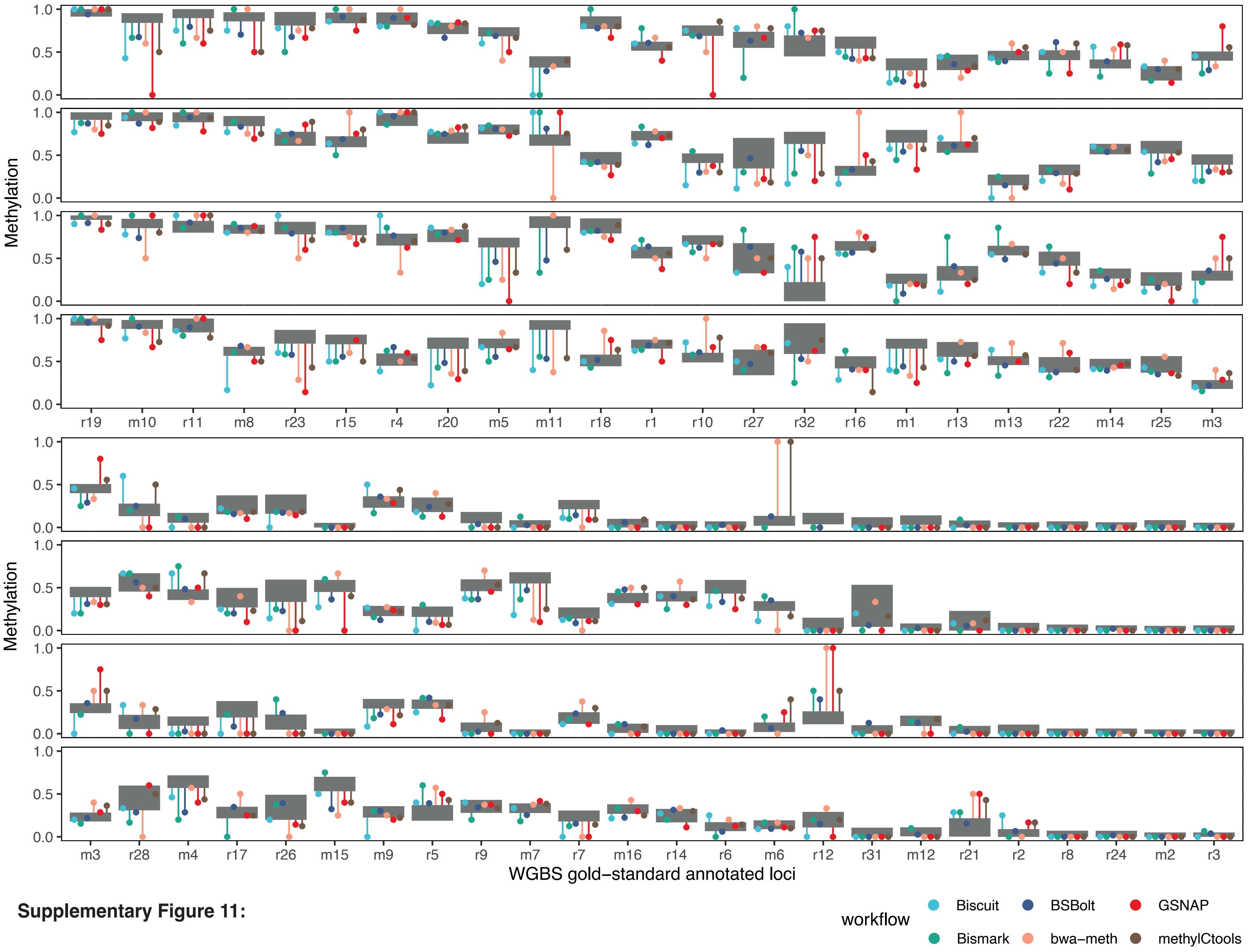

Supplementary Figure 11:

Supplementary Figure 12

Same as Supplementary Figure 10, but for the Swift protocol.

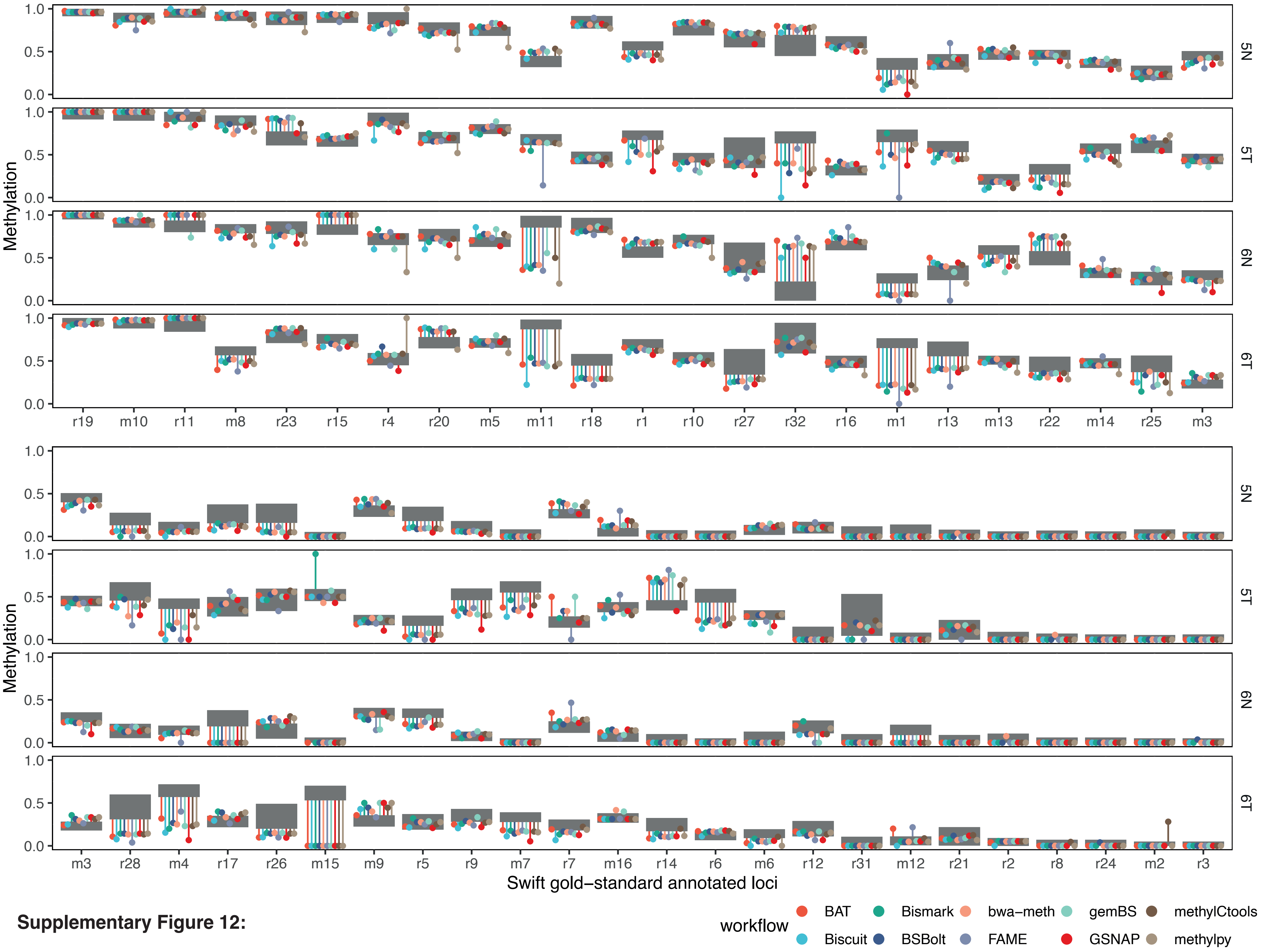

Supplementary Figure 12:

Supplementary Figure 13

Same as Supplementary Figure 10, but for the T-WGBS protocol.

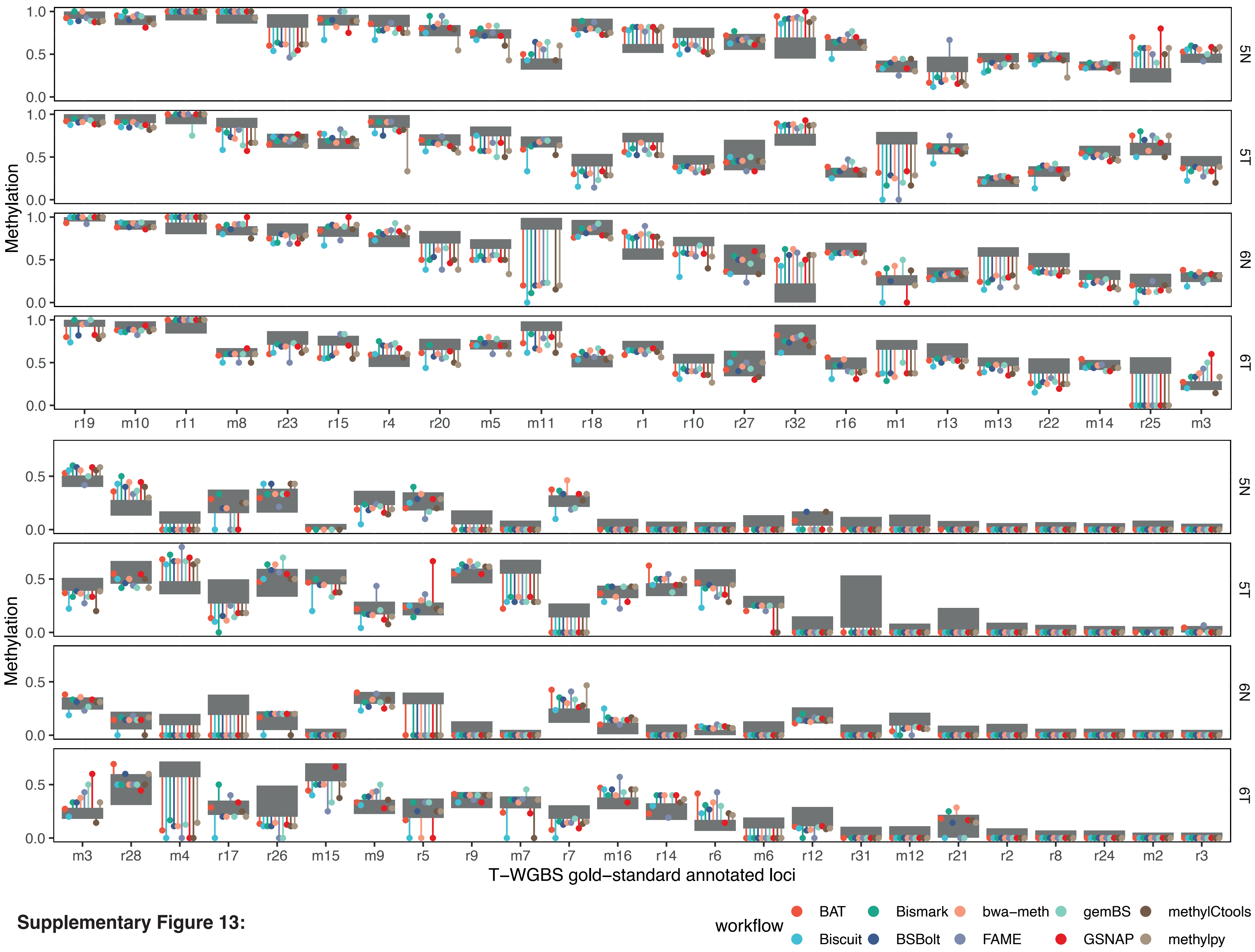

Supplementary Figure 13:

Supplementary Figure 14

Same as Supplementary Figure 10, but for the PBAT protocol.

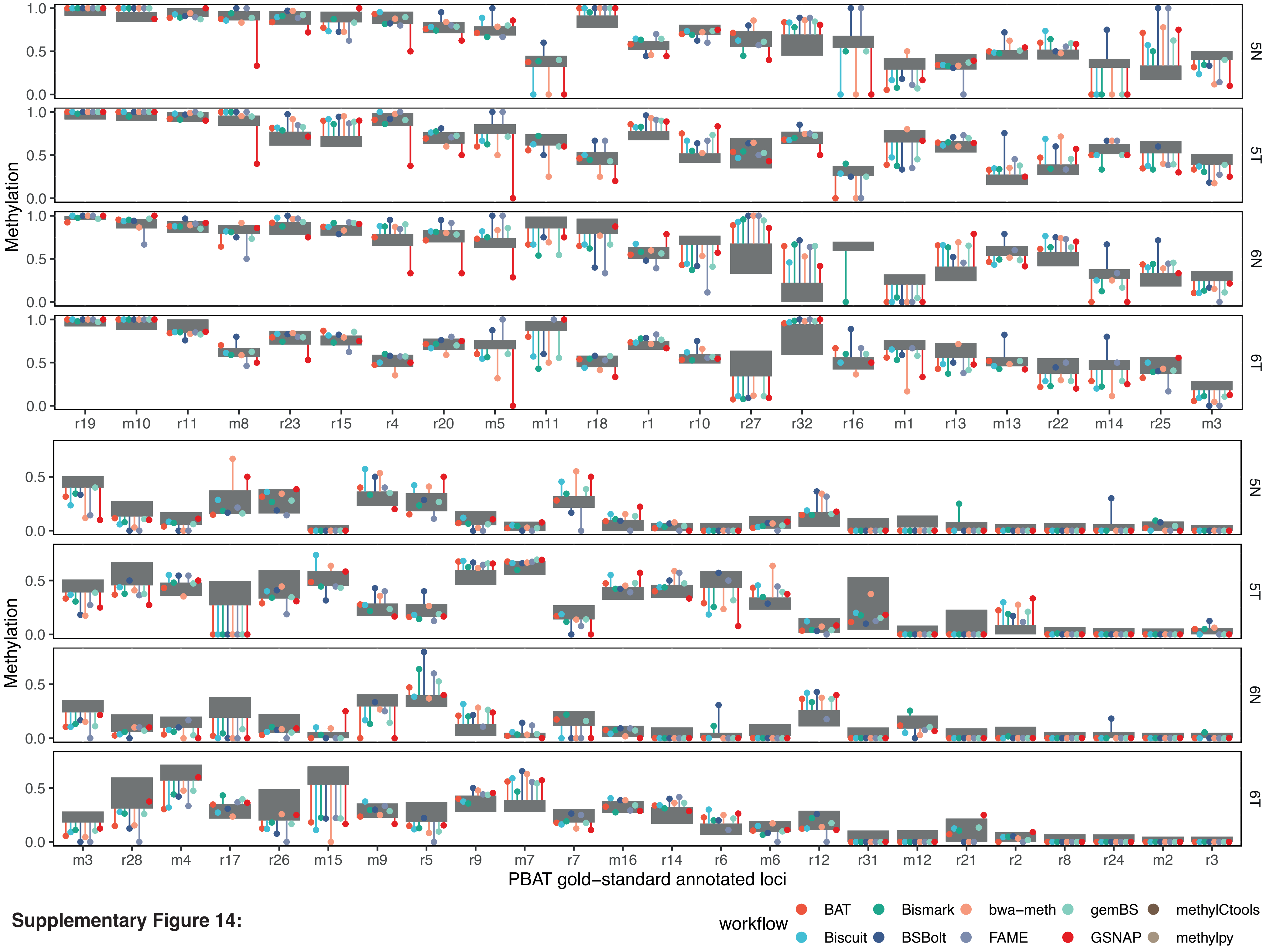

Supplementary Figure 14:

Supplementary Figure 15

Same as Supplementary Figure 10, but for the EM-seq protocol.

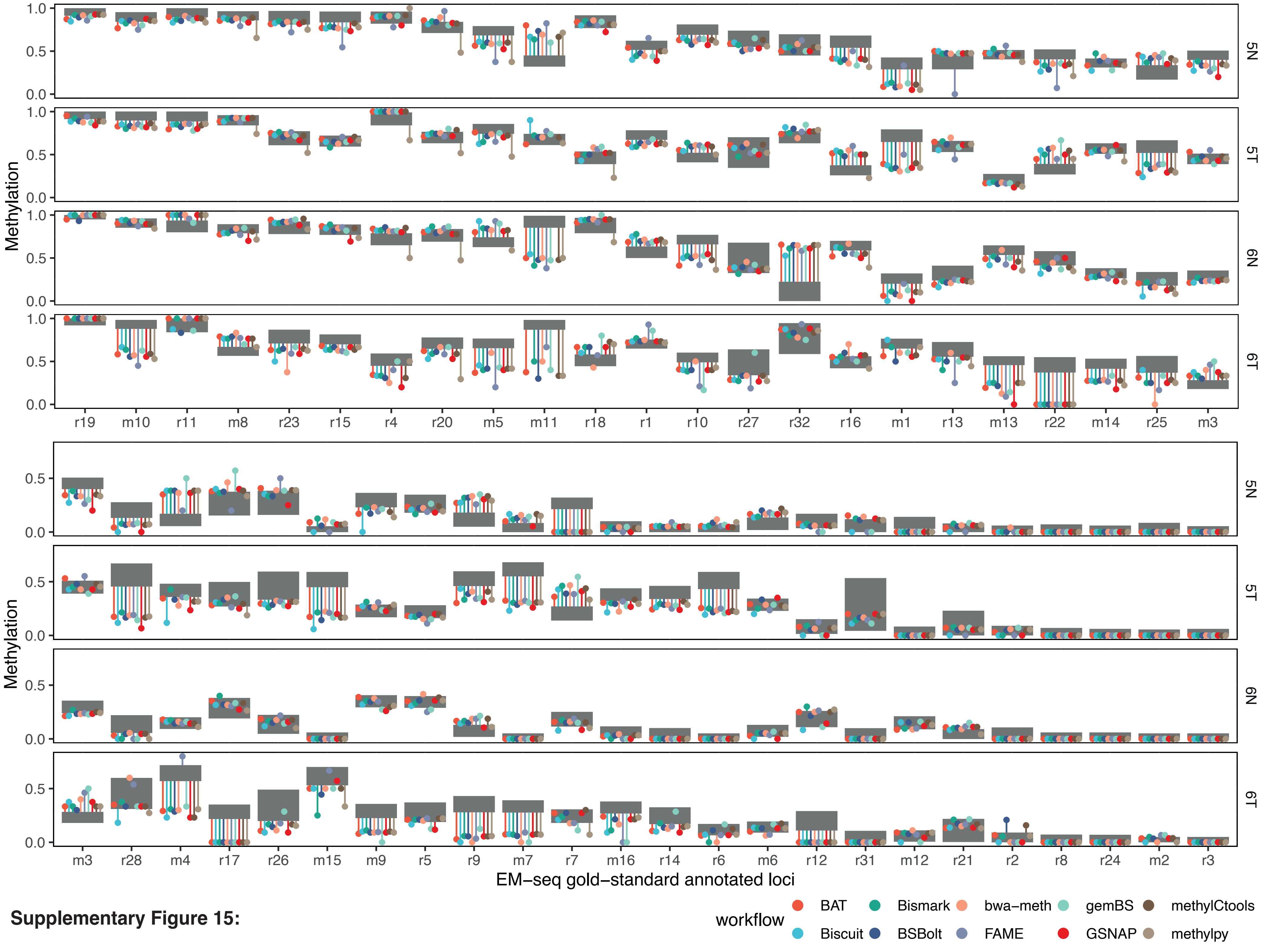

Supplementary Figure 15:

Supplementary Figure 16

Deviations from gold standard consensus corridors (an extension of Figure 5b). The deviation from the consensus corridors (CC) of the four protocols for all samples combined, while the WGBS data is shown in Figure 5b. The pie charts on the left illustrate the proportion of data points falling outside versus inside the CC. On the right, the box plot displays the distribution of deviations, excluding data points within the CC. The columns labeled 'N/T' indicate whether the data pertains to normal or tumor samples, respectively. Positive values indicate prediction above the CC, while negative values indicate predictions below the CC.

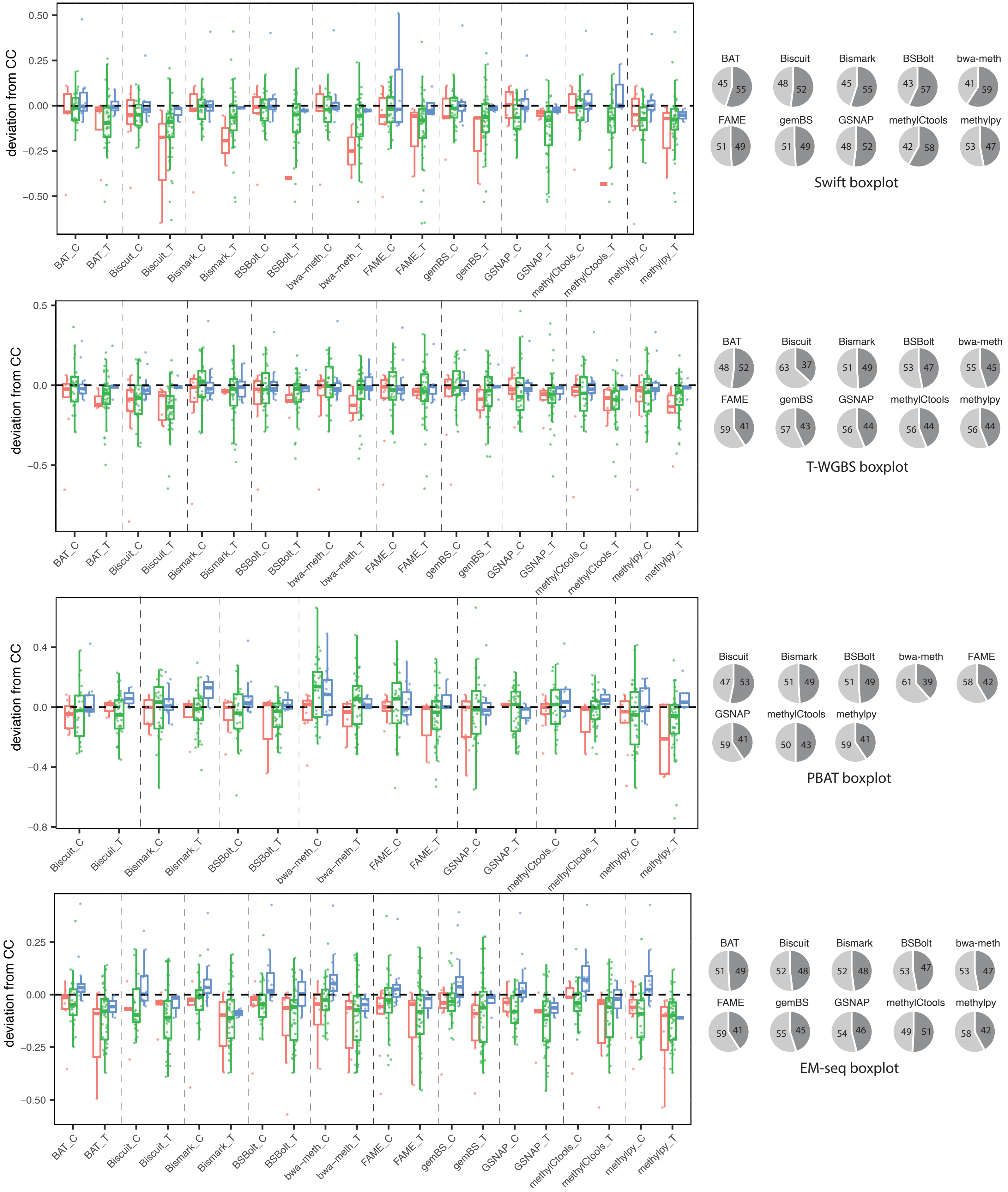

Supplementary Figure 16:

Supplementary Figure 17

Example of a software bug resulting in erroneous methylation calls. In the case of the normal sample from WGBS patient 5, locus r23, *Methylpy* detects lower methylation beta values compared to other workflows. From (a), it can be observed that *Methylpy* determines lower beta values, and upon alignment, it is evident that both workflows have the same number of aligned reads. In (b), we observed that the intermediate BAM sequence and final sequence from *Methylpy* (3rd and 4th row) do not match raw read (1st) and BSBolt (2nd row). *Methylpy* employs a three-letter method that carries out in silico conversion of reads before alignment. In the methylation calling step, the converted reads must be restored to their original sequences. These observations are suggesting of an implementation error in the simulated bisulfite conversion.

a.

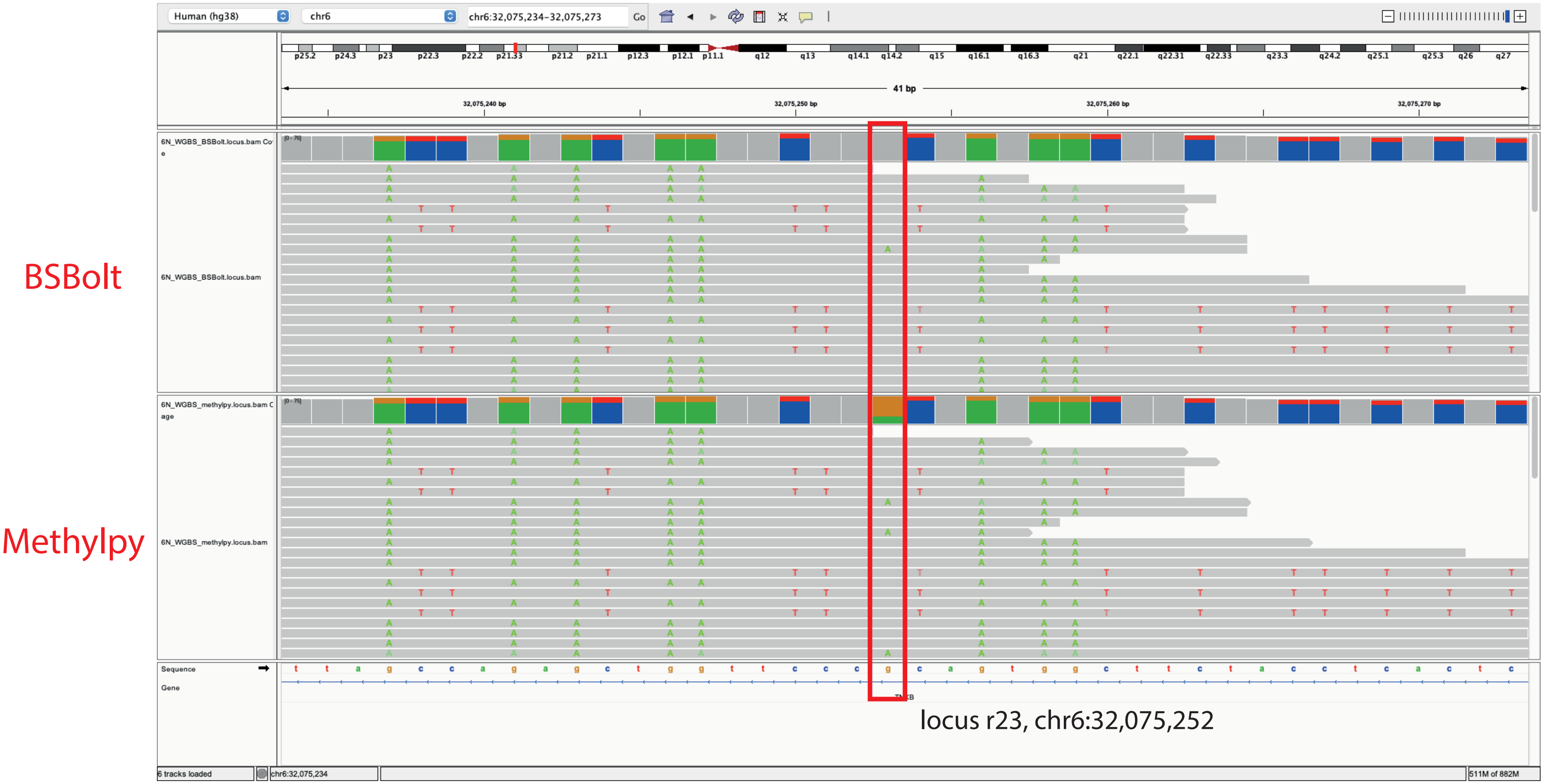

b.

(read name: ST-E00335:86:HWFCLCCXX:6:1211:19229:66409)

```
raw_19229:66409_r1_reverse_complement     ACATTAACCAAAACTAATTCCCGCAATAACTTCTACCTCACTCAAAATAAAATCCAAATC 60
bsbolt_alignment_r1                       ACATTAACCAAAACTAATTCCCGCAATAACTTCTACCTCACTCAAAATAAAATCCAAATC 60
methylpy_intermediate_reverse_r1          ACATTAACCAAAACTAATTCCCACAATAACTTCTACCTCACTCAAAATAAAATCCAAATC 60
methylpy_alignment_r1                     ACATTAACCAAAACTAATTCCCACAATAACTTCTACCTCACTCAAAATAAAATCCAAATC 60
                                          ********************** *************************************
```

Supplementary Figure 17:
